# Epidemiology of fractures in children with cerebral palsy: a Swedish population-based registry study

**DOI:** 10.1186/s12891-022-05813-9

**Published:** 2022-09-15

**Authors:** Gustaf Linton, Gunnar Hägglund, Tomasz Czuba, Ann I. Alriksson-Schmidt

**Affiliations:** 1 Faculty of Medicine, Lund University, Lund, Sweden; 2 Department of Clinical Sciences Lund, Orthopedics, Lund University, Skåne University Hospital, 221 85 Lund, Sweden

**Keywords:** Cerebral palsy, Fractures, Children, Total population

## Abstract

**Background:**

Children with cerebral palsy (CP) form a heterogeneous group and may have risk or protective factors for fractures compared with typically developing children. The fracture sites may also differ from those of children who do not have CP. We analyzed the fracture epidemiology in a total population of children with CP.

**Methods:**

This was a retrospective registry study based on data from the Swedish Cerebral Palsy Follow-Up Program (CPUP) and the Swedish National Patient Register. All children in the CPUP born in 2000–2015 were included. The Gross Motor Function Classification System (GMFCS) level, reported fractures, fracture site, and epilepsy diagnosis were recorded up to 2018. Hazards and hazard ratios were calculated for first-time fractures.

**Results:**

Of the 3,902 participants, 368 (9.4%) had at least one reported fracture. The cumulative risk of sustaining a fracture before age 16 years was 38.3% (95% confidence interval 33.9–42.4). The hazard for fracture was 7 times higher in children with epilepsy. The overall fracture incidence was not statistically significantly related to sex or GMFCS level. Fractures in the upper extremities were most prevalent in children with a lower GMFCS level, and femoral fractures were most prevalent in children at GMFCS level V. Most fractures occurred in early childhood and after 8 years of age.

**Conclusions:**

Children with CP were at similar risk of sustaining fractures as typically developing children, but the risk was higher in children with comorbid epilepsy. Fractures occurred in children at GMFCS levels I–III at sites similar to those for typically developing children; fractures in the upper extremities were the most frequent. Children at GMFCS levels IV or V and those with epilepsy were more likely to have a fracture in the lower extremities, and the femur was the most frequent site.

## Background

The diagnosis of cerebral palsy (CP) includes a wide range of motor impairments and associated conditions. Several factors may increase the risk of sustaining a fracture in children with CP, and some factors can reduce this risk. Gross motor function in people with CP is commonly classified using the Gross Motor Function Classification System (GMFCS), a five-level classification system in which people at levels I–III have varying degrees of walking ability, whereas those at level IV or V use a wheelchair for mobility [1].

Children with CP who walk may have an increased risk of falling because of impaired motor skills and balance. However, children with CP are less likely to participate in certain physical activities, which may be reflected in a lower incidence of fractures. In a study of the total population in southern Sweden, the fracture incidence was similar in children with CP and typically developing children [2]. However, fracture sites were not analyzed in that study.

Children who do not walk and those with nutritional deficiencies often have reduced bone mineral density (BMD), which is associated with an increased fracture risk. Epilepsy is also associated with an increased risk of fractures. This may be due to epilepsy itself but also to reduced BMD caused by antiepileptic drugs. The most common explanation is that antiepileptic drugs induce increased catabolism of vitamin D in the liver but also that it affects intestinal absorption of calcium and inhibits cellular response to parathyroid hormone [3]. Henderson et al. [4] reported osteopenia in the distal femur in 77% of 117 young people aged 2–19 years with CP at GMFCS levels III—V. Fractures occurred in 26% of the children who were older than 10 years, and the femur was the most common fracture site. Epilepsy was significantly associated with lower BMD and a higher risk of fractures [4]. These fragility fractures are painful and sometimes difficult to diagnose due to indistinct symptoms and the fact that these individuals sometimes have difficulty communicating their pain.

The purpose of this study was to analyze the incidence of fractures and the panorama of fracture sites according to age, sex, GMFCS level, and epilepsy status in the total population of children with CP aged 0–18 years in Sweden.

## Methods

This was a Swedish registry study based on data merged from the combined Swedish Cerebral Palsy Follow-Up Program (CPUP) and the National Patient Register (NPR). CPUP started in 1994 and, since 2006, has included more than 95% of all children and adolescents with CP in Sweden born in 2000 or later [5]. The NPR is maintained by the Swedish National Board of Health and Welfare and contains information about all inpatient and outpatient contacts from all hospitals in Sweden [6]. Data from CPUP and NPR were merged using each person's 10-digit national registration number, which is unique for every individual who legally resides in Sweden.

A child in Sweden suspected of having CP is offered the opportunity to participate in CPUP. After the age of 4 years, the diagnosis of CP is confirmed by a neuropediatrician, and children who are found not to have CP discontinue the program [6]. The diagnostic criteria developed by the Surveillance of Cerebral Palsy in Europe are used to diagnose CP [7]. Children with postneonatal causes of CP that occur before the second birthday are included. The GMFCS level is classified by the child’s physiotherapist. All children in CPUP born in 2000–2015 whose information on sex and GMFCS level was available were included in this study. Data about fractures, fracture site according to the International Classification of Diseases Version 10 (ICD-10), and reported epilepsy diagnosis were extracted from the NPR.

All hospital visits up to December 31, 2018 were recorded. For children who had died, their total years of life were calculated by subtracting the date of birth from the date of death.

### Statistical analysis

Descriptive data are presented as absolute numbers and percentages for categorical variables. Pearson’s χ^2^ test was used to analyze the presence of fractures (yes/no) according to sex, epilepsy status, and GMFCS level. *P*-values of ≤ 0.05 were considered to be significant. The fracture sites were classified according to sex, epilepsy status, and GMFCS level, and are presented descriptively as numbers and percentages.

Survival analysis was performed by fitting a flexible parametric survival model (stmp2 and standsurv commands [8]) using epilepsy as a time-dependent covariate (when all fractures is outcome) and sex (fractures in the upper extremities) because of nonproportional hazard ratios (HRs) and then adjusting for sex (all fractures and fractures in the lower extremities) and GMFCS level. The survival curves represent standardized hazard curves along with the corresponding HRs. Only first-time fractures were considered events. Stata/SE (v 15.1; StataCorp LLC) was used for the statistical analyses.

## Results

In total, 3,902 participants and 32,156 recorded hospital visits during the years 2000–2015 were included. The distributions of sex, epilepsy status, and birth year by GMFCS level are presented in Table 1.

**Table 1:** Characteristics of the children with cerebral palsy included in the study

	**Gross Motor Function Classification System level**						**Total**
	**I**	**II**	**III**	**IV**	**V**	**Missing**	
**Boys**	1,001	306	231	320	313	80	2,251
**Girls**	751	215	149	208	250	78	1,651
**Epilepsy** (% of children with epilepsy)	300 (17.1)	149 (28.6)	116 (30.1)	249 (47.2)	431 (76.6)	46 (29.1)	1,291 (33.1)
**Birth year**							
**2000–2004**	560	170	119	173	183	40	1,245
**2005–2010**	752	217	142	218	229	82	1,640
**2011–2015**	440	134	119	137	151	36	1,017
**Total**	1,752	521	380	528	563	158	3,902

In total, 436 fractures were identified in 368 children (9.4% of the population) (Table 2). Children with CP and a diagnosis of comorbid epilepsy had a significantly higher percentage of fractures (13.9%) (χ^2^ = 44.4, df = 1, *P* < 0.001) than those who did not have a diagnosis of epilepsy. The fracture incidence was not statistically significantly related to sex or GMFCS level. The cumulative risk of sustaining a fracture before 16 years of age was 38.3% (95% confidence interval (CI) 33.9–42.4).

**Table 2:** Fractures in children with cerebral palsy classified according to sex, GMFCS level, epilepsy, and age

	**Number of children with fracture, n (%)**	**Number of fractures**	**Total number of children**
Boys	199 (8.8)	238	2,251
Girls	169 (10.2)	198	1,651
GMFCS level		436	
I	168 (9.6)	190	1,752
II	49 (9.4)	69	521
III	29 (7.6)	31	380
IV	44 (8.3)	55	528
V	58 (10.3)	68	563
Missing data	20 (12.6)	23	158
Epilepsy	179 (13.9)	213	1,291
No epilepsy	189 (7.2)	223	2,611
Birth year			
2000–2004	215 (17.3)	258	1,245
2005–2010	126 (7.7)	142	1,640
2011–2015	27 (2.7)	36	1,017
Total	368 (9.4)	436	3,902

*GMFCS* Gross Motor Function Classification System

**Fig. 1:**
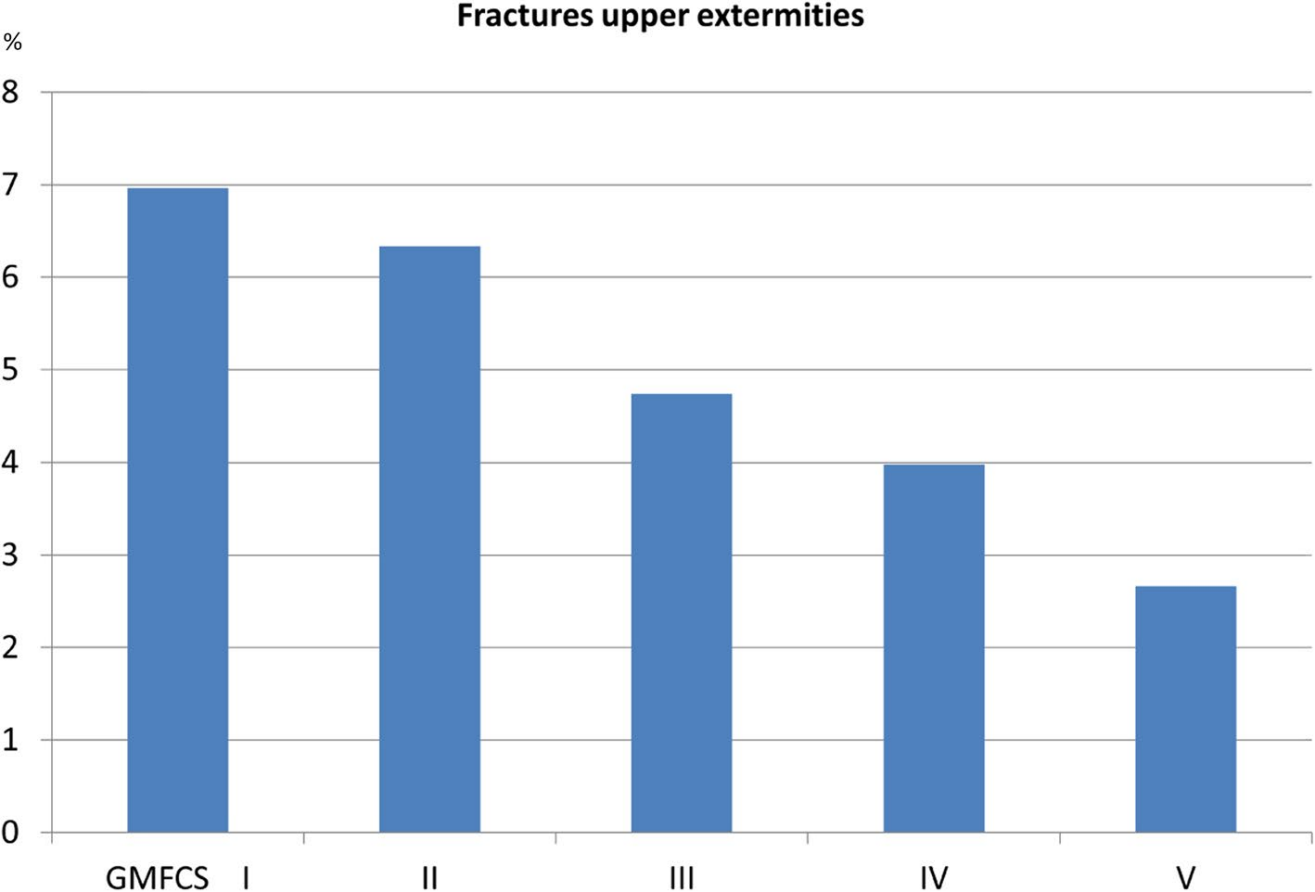
Percentages of fractures in the upper extremities grouped according to the Gross Motor Function Classification System (GMFCS) level

**Fig. 2:**
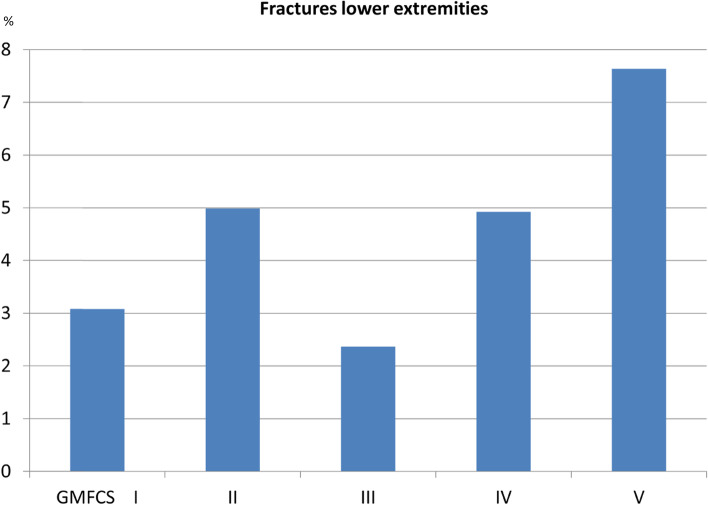
Percentages of fractures in the lower extremities grouped according to the Gross Motor Function Classification System (GMFCS) level

The most common fracture sites were the humerus or clavicle (2.3% of all children included; 20.4% of all fractures), radius–ulna (1.9%; 17.4%), and tibia–fibula (1.8%, 16.3%) (Table 3). Upper-extremity fractures were most common in children at GMFCS level I and the rate decreased with increasing GMFCS level (Fig. 1). Lower-extremity fractures were most common in children at GMFCS level V and least common in children at GMFCS level I or III (Fig. 2). Spinal and pelvic fractures were rare in all GMFCS-levels. Fracture of the femur was the most common fracture site among children at GMFCS level V and comprised 32% of all fractures in children at GMFCS level V but was among the least common fracture site in children at all other GMFCS levels.

**Table 3:** Fractures in children with cerebral palsy according to fracture site, sex, GMFCS level, epilepsy status, and age

	**Upper extremities**			**Thoracic spine**	**Lower extremities**			**Head**	**Total**
	**Humerus—clavicula**	**Radius–ulna**	**Hand**		**Femur**	**Tibia–fibula**	**Foot**		
**Boys**	48 (20)	39 (16)	34 (14)	2 (1)	20 (8)	35 (15)	33 (14)	27 (11)	238
**Girls**	41 (21)	37 (19)	20 (10)	3 (2)	21 (11)	36 (18)	24 (12)	16 (8)	198
**GMFCS level**									
**I**	44 (23)	50 (26)	28 (15)	1 (1)	4 (2)	24 (13)	26 (14)	13 (7)	190
**II**	14 (20)	11 (16)	8 (12)	3 (4)	4 (6)	13 (19)	9 (13)	7 (10)	69
**III**	4 (13)	7 (23)	7 (23)	0	1 (3)	5 (16)	3 (10)	4 (13)	31
**IV**	10 (18)	5 (9)	6 (11)	1 (2)	6 (11)	13 (24)	7 (13)	7 (13)	55
**V**	10 15)	1 (1.5)	4 (6)	0	22 (32)	11 (16)	10 (15)	10 (15)	68
**Missing data**	7	2	1	0	4	5	2	2	23
**Epilepsy**	38 (18)	29 (14)	24 (11)	1 (0.5)	31 (15)	33 (15)	28 (13)	29 (14)	213
**No epilepsy**	51 (23)	47 (21)	30 (13)	4 (2)	10 (4)	38 (17)	29 (13)	14 (6)	223
**Birth year**									
**2000–2004**	46 (18)	50 (19)	37 (14)	3 (1)	25 (10)	41 (16)	36 (14)	20 (8)	258
**2005–2010**	38 (27)	23 (16)	16 (11)	0 (0)	9 (6)	24 (17)	16 (11)	16 (11)	142
**2011–2015**	5 (14)	3 (8)	1 (3)	2 (6)	7 (19)	6 (17)	5 (14)	7 (19)	36
**Total**	89 (20)	76 (17)	54 (12)	5 (1)	41 (9)	71 (16)	57 (13)	43 (10)	436

Data are expressed as n (%)

*GMFCS* Gross Motor Function Classification System

### Overall fracture prevalence

**Fig. 3:**
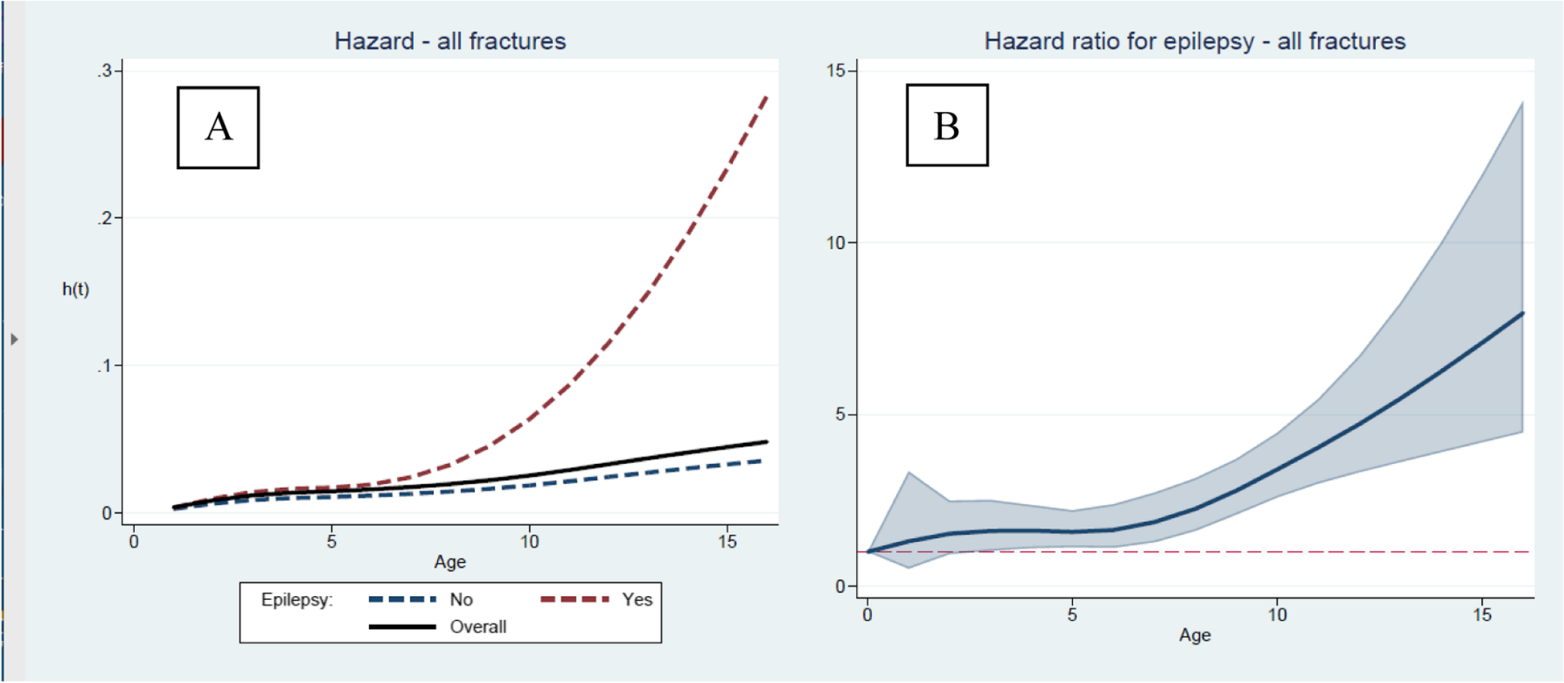
**A** Hazard of fracture in all children, children with and without epilepsy after adjusting for sex and GMFCS level. **B** Hazard ratio for epilepsy adjusted for sex and GMFCS level

The proportional-hazards model was violated by the epilepsy variable. We therefore modeled the hazard analysis for sustaining a first-time fracture by allowing the hazard to change over time and adjusting for sex and GMFCS level. The nonproportionality of the hazard for epilepsy is best presented graphically. The overall hazard and the hazard for children with and without epilepsy are presented in Fig. 3A, and the HRs for epilepsy in Fig. 3B. The hazard for fracture was almost equal in children with and without epilepsy up to 6 years of age but then increased to 23% for children with epilepsy at 15 years of age compared with 3% in those without epilepsy (Fig. 3A). The HRs for fractures did not differ significantly according to sex or GMFCS level (Table 4).

**Table 4:** Hazard ratios (HRs) for fracture in children with cerebral palsy in relation to sex and GMFCS level

Variable	HR	*P*-value	95% CI
Boys*	0.91	0.39	0.74 – 1.12
GMFCS level**			
II	1.04	0.77	0.78 – 1.41
III	0.69	0.06	0.46 – 1.02
IV	0.77	0.12	0.56 – 1.07
V	0.86	0.36	0.63 – 1.18

*GMFCS* Gross Motor Function Classification System, *CI* confidence interval

^*^ Girls as reference

^**^ GMFCS level I as reference

### Fractures in the upper and lower extremities

**Fig. 4:**
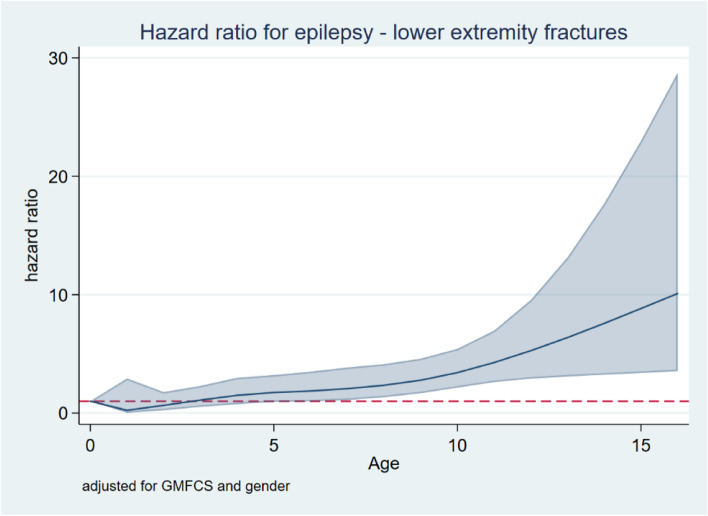
Hazard ratio for lower-extremity fracture in children with epilepsy after adjusting for GMFCS and sex

The proportional-hazards model was violated by epilepsy for lower-extremity fractures and by epilepsy and sex for upper-extremity fractures. For lower-extremity fractures, the HR increased in children with epilepsy after 8 years of age and was 8.8 (95% CI 3.4–23) at 15 years of age (Fig. 4). The HRs were stastiistically significantly higher for girls and for children at GMFCS level V (Table 5).

**Table 5:** Hazard ratios (HRs) for fracture in children with cerebral palsy in the lower extremities in relation to sex and GMFCS level

Variable	HR	*P*-value	95% CI
Boys*	0.70	.04	0.50 – 0.98
GMFCS level**			
II	1.43	.16	0.87 – 2.36
III	0.77	.47	0.38 – 1.57
IV	1.29	.33	0.77 – 2.16
V	1.89	.01	1.19 – 3.02

*GMFCS* Gross Motor Function Classification System, *CI* confidence interval

^*^ Girls as reference

^**^GMFCS level I as reference

**Fig. 5:**
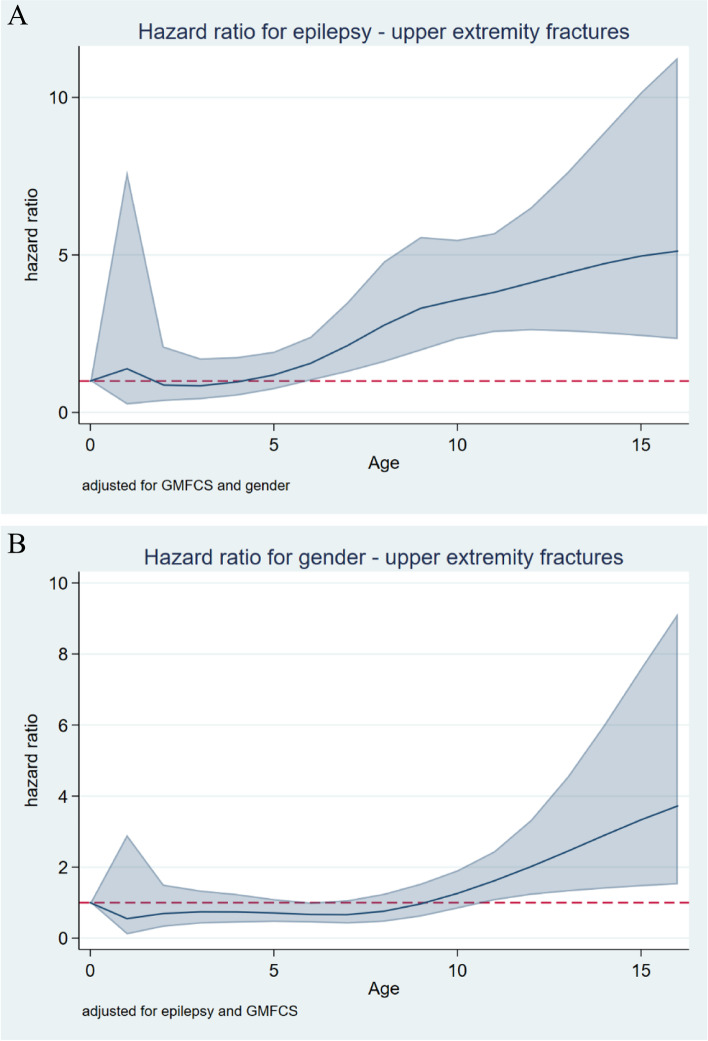
**A** Hazard ratio for upper-extremity fracture in children with cerebral palsy with (blue line) and without (red line) epilepsy after adjusting for sex and GMFCS level. **B** Hazard ratio for upper-extremity fracture in boys with cerebral palsy (blue line) relative to girls (red line) after adjusting for sex and epilepsy status

For upper-extremity fractures, the HR increased in children with comorbid epilepsy after 5 years of age and was 5 (CI 2.4–10.2) at 15 years of age (Fig. 5A). The HR for boys relative to girls increased after 9 years of age and was 3.3 (CI 1.5–7.6) at 15 years of age (Fig. 5B). The HR was statistically significantly lower for children at higher GMFCS levels (Table 6).

**Table 6:** Hazard ratios (HRs) for fracture in children with cerebral palsy in the upper extremities in relation to GMFCS level

GMFCS level*	HR	*P*-value	95% CI
II	0.73	.15	0.47 – 1.12
III	0.59	.049	0.34 – 1.00
IV	0.45	.002	0.27 – 0.75
V	0.34	< .001	0.19 – 0.60

*GMFCS* Gross Motor Function Classification System

^*^GMFCS level I as reference

**Fig. 6:**
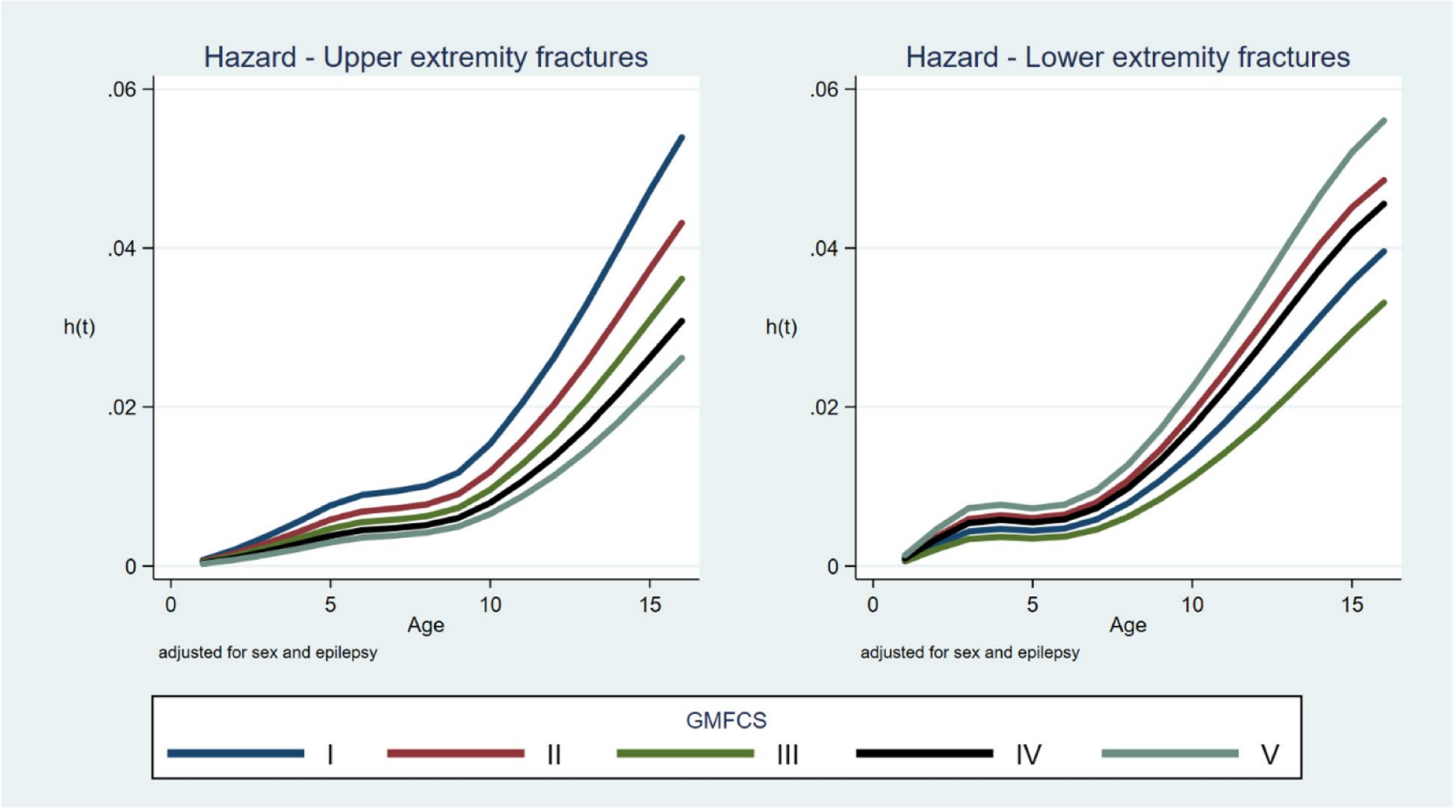
Hazard ratio for fracture in the upper (left) and lower (right) extremities related to GMFCS level and age after adjusting for sex and epilepsy

The hazard for fracture related to age in all GMFCS levels was highest before the age of 3 years for the lower extremities and 6 years for the upper extremities. The risk was also highest after the ages of 6 years for the lower extremities and 8 years for the upper extremities (Fig. 6). The order of fracture hazard according to GMFCS level differed between fractures in the upper and lower extremities.

## Discussion

The main findings of this study were that children with CP had a 38% cumulative risk of sustaining a fracture up to 16 years of age. The HR for fracturing at least once was 7 times higher at 15 years of age in children with a comorbid diagnosis of epilepsy. Fracture in the upper extremities was most prevalent in children at the lower GMFCS levels, and fracture in the femur was most prevalent in children at GMFCS level V. The overall fracture incidence did not differ according to sex or GMFCS level.

The percentage of children who experienced fracture up to the age of 15 years is similar to that reported for the total population of children in Sweden. Hedström et al. reported a 34% cumulative fracture risk before 17 years of age in northern Sweden [9]. Tiderius et al. reported a fracture risk of 42% in boys and 26% in girls before 16 years of age in southern Sweden [9, 10]. In contrast to other population-based studies, our analyse did not show a stastistically significant sex difference in the overall fracture incidence for children with CP.

The panorama of fracture sites for children at GMFCS levels I–III is similar to the distribution in the total population, and most fractures occur in the upper extremities [9]. Falls are the most common cause of fractures in typically developing children, usually in connection with play and sports [9]. We do not have information on the etiologies of fractures in our study, but it is likely that falls were the predominant cause for children at GMFCS levels I–III. Poorer balance and reduced muscle strength may make these children more prone to falling.

Fractures among children at GMFCS level IV or V were more frequent in the lower extremities, and femoral fractures were the most frequent site in children at GMFCS level V. Fracture of the femur in children at GMFCS level V is often caused by minor trauma and correlates with low body mass index, nutritional problems, epilepsy treatment, and contractures [4, 11, 12]. Fracture of the femur can occur in association with skeletal surgery, postoperative plaster treatment, or extraction of osteosynthesis material [13].

Presedo et al. [13] studied a cohort of 156 children with CP who were treated for fractures at a tertiary center. The authors reported a high percentage of cases (55%) for which the cause of the fracture was unknown and that the diagnosis was delayed in 46% of all fractures. Bajelidze et al. [14] examined 45 individuals with CP who were unable to communicate and with suspected pain as identified using whole-body scintigraphy. In 10 of these individuals (22%), the likely cause of the pain was an undiagnosed fracture. These studies show that fractures are probably missed or underdiagnosed in people who have difficulties communicating or with intellectual disability.

Skull and head fractures accounted for 10% of all fractures in children with CP. The corresponding percentage among typically developing children is 1% [9]. Sixteen of the 43 fractures occurred before the age of 2 years and may have been the actual cause of a postnatal CP, which may partially explain the high percentage of skull fractures.

No child in the study experienced a pelvic fracture and spinal fractures were rare. Previous studies have also reported a low frequency of spinal fracture in children with CP [4, 12, 13]. Studies of BMD in children with CP have shown a statistically significantly smaller reduction in BMD in the spine than in the lower extremities [4, 11]. One explanation may be that sitting leads to a greater reduction in loading of the lower extremities than the spine.

The HR for fractures in children with epilepsy increased after 5–8 years of age. Souverein et al. [3] reported that the fracture incidence increased with cumulative duration of exposure to antiepileptic drugs, which may explain the increasing HR with age in our study.

Prevention and treatment of low bone density are important for reducing the incidence of fractures, especially in children at GMFCS level IV or V and in those treated for epilepsy. Physical activity [15], weight-bearing exercise [16], standing on a vibrating platform [17], vitamin D [18], and adequate nutritional intake [2, 4] have been reported to have positive effects on BMD. Studies have shown a good effect of bisphosphonate treatment on BMD. Henderson et al. [19] observed that the distal femoral metaphyseal BMD increased by 89% in six children with CP treated with intravenous pamidronate for 18 months compared with a 9% increase in the placebo group.

The osteoporosis care pathway of the American Academy of Cerebral Palsy and Developmental Medicine recommends prevention of individuals at risk for osteoporosis with adequate intake of calcium and vitamin D as well as weight bearing activities. Investigation of BMD and possibly prevention with bisphosphonates are proposed if an individual with CP has a fragility fracture [20]. Based on our results, it should be considered whether individuals with CP at GMFCS level V and comorbid epilepsy should be included in the group that should be further investigated and treated with bisphosphonates, even before they have had a fracture. Further investigation of this approach to intervention for this subgroup will be beneficial.

One limitation of this study is that we did not have data on the fracture etiology or on the epidemiology of repeated fractures. Although these have been reported to the NPR, it was not possible to determine whether repeated reports of fracture diagnoses referred to a new fracture or new visits because of the original fracture. It would have been useful to have more detailed information about the duration and dosage of anticonvulsant medication, but that information was not available. Another limitation is that we were unable to study the total number of fractures for each child, which would have been useful information.

## Conclusions

Children with CP had a similar incidence of fractures as typically developing children. However, the incidence was 7 times higher in children with CP and comorbid epilepsy. Children at GMFCS levels I–III had a similar fracture panorama as that of typically developing children, and most fractures occurred in the upper extremities. Children at GMFCS level IV or V and those with comorbid epilepsy experienced fractures more often in the lower extremities, and the femur was the most common fracture site.

## Data Availability

The CPUP database is owned by Skane Regional Council (Region Skane, 29,189 Kristianstad, Sweden) and the NPR database by the National Board of Health and Welfare (Socialstyrelsen, 10,630 Stockholm, Sweden). The datasets used and/or analysed during the current study are available from the corresponding author on reasonable request and with permission of Skane Regional Council and the National Board of Health and Welfare.

## Abbreviations

BMD: Bone mineral density
CI: Confidence interval
CP: Cerebral palsy
CPUP: The Swedish Cerebral Palsy Follow-Up Program
GMFCS: Gross Motor Function Classification System
HR: Hazard ratio
ICD: International Classification of Diseases
NPR: National Patient Register
